# DNA Damage Inducible Protein 1 is Involved in Cold Adaption of Harvested Cucumber Fruit

**DOI:** 10.3389/fpls.2019.01723

**Published:** 2020-01-24

**Authors:** Bin Wang, Guang Wang, Shijiang Zhu

**Affiliations:** ^1^ Guangdong Province Key Laboratory of Postharvest Science of Fruits and Vegetables/Key Laboratory of Biology and Germplasm Enhancement of Horticultural Crops in South China, Ministry of Agriculture, College of Horticulture, South China Agricultural University, Guangzhou, China; ^2^ College of Ying-Tong Agricultural Science and Engineering, Shaoguan University, Shaoguan, China

**Keywords:** DNA damage response, cold acclimation, chilling tolerance, H_2_O_2_, cucumber fruit

## Abstract

Chilling stress can cause cellular DNA damage, affecting the faithful transmission of genetic information. Cold acclimation enhances chilling tolerance, but it is not clear that the process of cold adaption involves DNA damage responses, as cold acclimation does not form real chilling stress. Here we showed with cucumber fruit that pre-storage cold acclimation (PsCA) reduces chilling injury and upregulates DNA damage inducible protein1 (*CsDDI1*), suggesting that the chilling tolerance induced by cold acclimation involves *CsDDI1* transcription. Application of nitric oxide (NO), abscisic acid (ABA) or H_2_O_2_ biosynthesis inhibitor before PsCA treatment downregulates *CsDDI1* and aggravates chilling injury, while H_2_O_2_ generation inhibition plus exogenous NO or ABA application before PsCA treatment restores chilling tolerance, but does not restore *CsDDI1* expression, suggesting H_2_O_2_ plays a crucial role in triggering cold adaption. *CsDDI1* overexpression *Arabidopsis* lines show faster growth, stronger chilling tolerance, lower reactive oxygen species levels, enhanced catalase and superoxide dismutase activities and higher expression of nine other *Arabidopsis* defense genes under chilling stress, suggesting *CsDDI1* strengthens defenses against chilling stress by enhancing antioxidant defense system. Taken together, *CsDDI1* positively regulates chilling tolerance induced by cold acclimation in cucumber. In addition, H_2_O_2_ is involved in initiation of cold acclimation. While *CsDDI1* upregulation requires H_2_O_2_ as a key signaling molecule, the upregulation of *CsDDI1* activates an antioxidant system to reduce biotoxic accumulation of H_2_O_2_ and helps in DNA repair.

## Introduction

Plants have evolved the ability to cope with various environmental stresses to ensure survival and proliferation, including solar UV and ionizing radiation, chemical mutagens, heavy metals, droughts, heat, pathogenic attacks, and chilling. Although different environmental stresses may cause different disorders or symptoms in plants, they can all cause cellular DNA damage (Durrant et al., 2007; Gichner et al., 2008; Ciccia and Elledge, 2010; Achary et al., 2012; Cvjetko et al., 2014; Roy, 2014; Daghino et al., 2016; Ding et al., 2016; Maric et al., 2017; Shim et al., 2018). DNA damage may result in changes to both the chemical and physical structures of DNA, which can seriously threaten the survival and the faithful transmission of genetic information in plants (Zhang et al., 2015a; Ding et al., 2016). For example, chilling stress leads to DNA damage in root stem cells and their early descendants in *Arabidopsis* (Hong et al., 2017).

The organisms may initiate responses to defend itself against DNA damage. DNA damage-inducible (DDI) proteins are usually related to plant defense responses and play important roles in DNA repair pathways (Maric et al., 2017). *Arabidopsis* DNA damage-inducible protein 1 (AtDDI1) participates in plant defense responses against abiotic stresses such as drought and salt by regulating the expression of defense genes (Ding et al., 2016).

DNA damage binding (DDB) proteins are involved in damage recognition in global genomic repair (GGR), a sub-pathway of nucleotide excision repair (NER) (Shuck et al., 2008). DDB may function to alter chromatin structure and recruit NER factors to DNA damage sites (Gillet and Scharer, 2006). AtDDB1 is involved in DNA damage protection; overexpression of *DDB1A* and *DDB2* increases UV resistance, while *ddb1a* knockout and *AtDDB2* loss of function lead to increased UV sensitivity (Al Khateeb and Schroeder, 2009; Biedermann and Hellmann, 2010).

DNA-damage repair/tolerance (DDR or DRT) proteins are members of the RecA protein family and are involved in RecA-mediated homologous recombination (HR), which may play a role specifically in the repair/reduction of abasic sites and DNA single-strand breaks in plants (Pang et al., 1992; Fujimori et al., 2014). *VvDRT100-L* overexpressing plants remain noticeably healthier under lethal UV radiation, suggesting that VvDRT100-L may enhance plants’ tolerance against UV (Fujimori et al., 2014).

One of the most challenging environmental stresses that a plant faces is chilling during its development. Fruits, in particular, either as reproductive organs (Valenzuela et al., 2017) or as commercial products (Wang et al., 2018a) are susceptible to chilling injury because of the large water content in their tissues. Therefore, chilling injury, which is usually caused by suboptimal non-freezing low temperature, is one of the leading factors that affects fruit quality and seed development.

The occurrence of chilling injury is often concomitant with the production of reactive oxygen species (ROS) (Asada, 2006; Zhang et al., 2016a). If the generated ROS are not scavenged in a timely manner, they may cause DNA damage and are one of the primary causes of DNA decay in plants (Roldan-Arjona and Ariza, 2009). On the other hand, ROS have been established as signal molecules in plant defense responses against abiotic stresses (Baxter et al., 2014; Lamotte et al., 2015; Farnese et al., 2016). H_2_O_2_ is the only stable ROS species in solution and can diffuse across cell membranes, making H_2_O_2_ a fit signaling molecule (Farnese et al., 2016).

Plants have evolved the ability to acquire chilling and freezing tolerance after being exposed to low non-freezing temperatures (Park et al., 2015; Jiang et al., 2017), a process referred to as cold acclimation (Thomashow, 1999). ROS, nitric oxide (NO) and abscisic acid (ABA) are essential for plant cold acclimation (Gusta et al., 2005; Shapiro, 2005; Zhao et al., 2009; Zhou et al., 2012; Yu et al., 2014). The elevated ROS concentrations result in the activation of nitrate reductase (NR) (Lin et al., 2012; Sewelam et al., 2016). The increased NR-mediated NO production in turn regulates NADPH oxidase activity and antioxidant systems, resulting in reduced H_2_O_2_ accumulation (Yun et al., 2011; Groß et al., 2013; Sevilla et al., 2015; Begara-Morales et al., 2016). ABA induces production of H_2_O_2_ and NO, which in turn induce the transcription/translation and activity of antioxidant enzymes (Zhou et al., 2005; Zhang and Wang, 2009). NO and ROS interact each other to regulate ABA biosynthesis and then to modulate stomatal closure (Sewelam et al., 2016). These suggest a subtle interaction among ROS, NO and ABA in the regulation of the defense responses of plants. However, it is unclear whether and how these three signal molecules are involved in the regulation of *CsDDI1* in response to cold stress.

We previously demonstrated that cold acclimation enhances chilling tolerance in cucumber fruit through the activation of antioxidant systems (Wang and Zhu, 2017; Wang et al., 2018b). Using proteomic analysis, we demonstrated that cold acclimation significantly increased CsDDI1 accumulation in cucumber (Wang et al., 2018a). However, it is not clear whether CsDDI1 plays a role in initiating cold acclimation. In the current study, we used physiological, biochemical and genetic approaches to show that cold acclimation significantly upregulates *CsDDI1* expression in a H_2_O_2_-mediated manner, which in turn upregulates a CsDDI1-activated antioxidant system to reduce biotoxic accumulation of H_2_O_2_ and to alleviate chilling injury.

## Materials and Methods

### Plant Materials and Treatment

Cucumber (*Cucumis sativus* L. cv Huaqing) fruit harvested at commercial maturity from a farm in Yinan County, Shandong Province, China, were transported to the laboratory within 24 h of harvest. All fruit were selected for uniform size and were free of blemishes, without mechanical damage and disease symptoms. During the years 2013 through 2017, three experiments were conducted, each repeated at least two times. The results presented here were from one set of experiments.

The first experiment was conducted to investigate the effects of pre-storage cold acclimation (PsCA) on chilling tolerance in relation to expression of DNA damage related genes. There were two treatments in this experiment: storage at 5°C (Control) and incubation at 10°C for 3 d followed by storage at 5°C (PsCA).

The second experiment was conducted to investigate the roles of endogenous abscissic acid (ABA) or nitric oxide (NO) in PsCA-induced tolerance and whether *CsDDI1* expression is regulated by endogenous NO and ABA. In this experiment, there were four treatments: Control, PsCA, TS (tungstate, ABA biosynthesis inhibitor)+PsCA, and L-NAME(L-nitro-arginine methyl ester, NO biosynthesis inhibitor)+PsCA. For the application of the combination treatments, the fruit were first sprayed with TS or L-NAME and then were air-dried at ambient temperature for 3 h before exposure to cold acclimation at 10°C.

The third experiment was conducted to unravel the relationships among H_2_O_2_, NO and ABA. In this experiment, there were five treatments: Control, PsCA, DPI (diphenylene iodonium, a NADPH oxidase inhibitor)+PsCA, DPI+ABA+PsCA, DPI+SNP (sodium nitroprusside, nitric oxide donor)+PsCA. For application of the combination treatments, the fruit were first sprayed with DPI, incubated in plastic bags for 3 h, and then air-dried at ambient temperature before the next treatment was applied. After the application of all reagents, the fruit were then cold acclimated at 10°C for 3 d.

Concentrations of chemicals used in the above experiments were as follows: TS at 50 μM, L-NAME 100 μM, ABA 100 μM, SNP 10 μM, DPI 10 μM (Zhang et al., 2015b; Liu et al., 2016). The solutions were applied in a fine mist until runoff to 90 fruit per treatment.

Each treatment was replicated three times, each time with 90 fruit. Following treatment, fruit were wrapped with perforated polyethylene film in the dark at 95% RH for 12 d of cold storage. Peel tissues from 3 fruit for each treatment were collected at 0 h (or 0 d, untreated), 3 h, 6 h, 12 h, 24 h, 48 h, 72 h (3 d) and every 2 days afterward. The peel tissues from each sample were then pooled and ground to powder in liquid nitrogen and stored at −80°C. Of the 90 fruit, 30 fruit of each treatment were labeled for observation of chilling injury severity and the rest for sampling.

### Evaluation of Chilling Injury, Secondary Disease, and Electrolyte Leakage

Chilling injury and secondary disease symptoms of the fruit surface were evaluated for 30 fruit for each replicate using a subjective scale of visual symptoms described previously (Liu et al., 2016). Chilling injury development was observed during storage at 5°C and secondary disease development was observed at ambient temperature (20°C) following 12 d of cold storage. Chilling injury or secondary disease severity scores range from 0 to 4, where 0 represents no pitting (chilling injury) or decay (secondary disease), 1 represents very slight pitting or decay (25% or less), 2 represents minor pitting or decay (25–50%), 3 represents medium pitting or decay (50-75%) and 4 represents severe pitting or decay (>75%). Chilling injury indices (CII) or secondary disease indices (SDI) were calculated using the following formula: ∑[pitting or decay scales (0–4) × number of corresponding fruit within each category]/total number of fruit evaluated.

Electrolyte leakage (EL) was measured as previously described (Liu et al., 2016). Briefly, the exocarp of cucumber fruit was separated with a vegetable peeler and 20 discs of cucumber peel tissue or *Arabidopsis* leaf were excised with a stainless steel cork borer (5 mm in diameter). The excised samples were rinsed three times with double distilled water before being incubated for 2 h at room temperature (25°C) in 25 ml of double distilled water. After 2 h of incubation, conductivity was measured using a conductance bridge (DDS-307, Leici Electron Instrument Factory, Shanghai). Total conductivity was determined after boiling the flasks for 30 min and cooling to room temperature. The EL was expressed as percentage of total conductivity.

### Analysis of Chlorophyll Fluorescence

Recently, maximal quantum yield of PSII (*Fv/Fm*) has been widely used to reflect chilling severity in harvested vegetables (Wang and Zhu, 2017; Fan et al., 2018; Tan et al., 2018). In the current study, *Fv/Fm* was measured using an imaging pulse amplitude modulated fluorometer (IMAG-K7, Walz, Germany) (Wang et al., 2018b). *Arabidopsis* plants were dark-adapted for 30 min to ensure sufficient opening of the reaction center before measurement. Minimal fluorescence (Fo) was measured during the weak measuring pulses and maximal fluorescence (Fm) was measured by a 0.8-s pulse light, and images for chlorophyll fluorescence were taken at the same time. *Fv/Fm* was calculated using the equation: *Fv/Fm* = (Fm − Fo)/Fm.

### RNA Extraction and Gene Expression Analysis

Total RNA from cucumber peels or *Arabidopsis* plants were extracted using Trizol reagent (Invitrogen, USA) according to the manufacturer’s instructions (Zhang et al., 2016b). The concentration and quality of total RNA was determined by spectrophotometry and visualized using 1.1% agarose gel. Genomic DNA was digested by RNase-free DNaseІ (Promega, USA) and the RNA remaining in the sample was then used to synthesize first-strand complementary DNA (cDNA). The cDNA was synthesized using iScript cDNA Synthesis Kit (Bio-Rad, USA) following the manufacturer’s instructions (Liu et al., 2016).

Quantitative real time PCR (qRT-PCR) was carried out using the SYBR Green PCR Master Mix (Bio-Rad, USA) as described previously (Wang and Zhu, 2017). The PCR reactions were performed by initial denaturation at 95°C for 15 min, followed by 40 cycles of 95°C for 15 s, 60°C for 20 s and 72°C for 30 s, using CFX96-Optics Module Real-Time PCR apparatus (Bio-Rad, USA). Expression values were normalized using cucumber *Actin* (*CsActin,* accession no. AB698859) or *Arabidopsis Actin* (*AtActin,* AGI code: AT3G12110). The relative expression levels of target genes were calculated using the formula (2^−△△Ct^) after normalization (Livak and Schmittgen, 2001). The specific primers (**Table S1**) were designed according to cDNA sequences using the Primer-BLAST tool of NCBI (National Center for Biotechnology Information) database.

### Isolation and Bioinformatics Analysis of *CsDDI1*

The Open Reading Frame (ORF) of *CsDDI1* was obtained from the Cucumber Genomic database (http://cucurbitgenomics.org/). cDNA from cucumber fruit peels was used as template for amplifying the full length of *CsDDI1.* The specific primers (forward, F1; reverse, F2) used for PCR amplification are listed in **Table S1**. Conditions for PCR amplification were as follows: 35 cycles of 94°C for 0.5 min, 60°C for 0.5 min and 72°C for 1 min, then a final step of 72°C for 10 min.

Gene sequence data was analyzed using the programs provided by BLASTN on the NCBI BLAST server (http://blast.ncbi.nlm.nih.gov/Blast.cgi). Molecular weight (MW) and isoelectric point (*pI*) of CsDDI1 were obtained using the ExPASy program (http://www.expasy.org/tools). Multiple alignments of amino acid sequences were analyzed using CLUSTALX (version 2.0) and mapped with the program DNAMAN (version 6.0). A phylogenetic tree of DDI1 from five plant species was constructed using the Neighbor-Joining (NJ) method in the MEGA5 program.

### Subcellular Localization of CsDDI1 Protein

The full-length CDS without the stop codon of *CsDDI1* was amplified by RT-PCR and was ligated into the C terminus of the green fluorescent protein (GFP) of a transient expression vector (*pCAMBIA2300-GFP*) between *Kpn I* and *Spe I* sites, driven by a cauliflower mosaic virus (CaMV) 35S promoter. The specific primers (F3 and F4) are listed in **Table S1**. The fusion constructs and control vector were electroporated into *Agrobacterium tumefaciens* strain GV3101 using Gene PulserXcellTM Electroporation Systems (Bio-Rad, USA) and then transformed into tobacco (*Nicotiana benthamiana*) leaf using the infiltration method. GFP fluorescence in tobacco leaf was observed 48 h after transfection using a fluorescence microscope (Zeiss Axioskop 2 Plus) (Wang et al., 2018a).

### Over-expression of CsDDI1 in *Arabidopsis*

To generate *35S::pCAMBIA 2300-CsDDI1* transgenic *Arabidopsis* plants, the coding sequence was subcloned into *pCAMBIA 2300* between the *Kpn I* and *Spe I* sites using T4 ligase, fused with 35S CaMV promoter. The construct *pCAMBIA 2300-CsDDI1* was then electroporated into GV3101 and transformed into *Arabidopsis* using the ﬂoral dip method (Zhang et al., 2006). The seeds were harvested and then sown onto MS selection medium containing kanamycin (50 μg/ml) for identification of the transgenic plants using the method as described previously (Zhang et al., 2016b). Two independent *35S::CsDDI1* overexpression lines were obtained. Plants were grown in growth chambers with a photoperiod of 16 h (13,000 lx)/8 h, the light/dark cycle at temperatures of 23/16°C. DNA and total RNA extracted from the kanamycin-resistant transformants of T1, T2 and T3 plants were used as templates to perform PCR using *CsDDI1* gene specific primers (F1 and F2, see **Table S1**). All PCR products were visualized on a 1.1% agarose gel containing 0.05‰ (v/v) gold view (Bio-Rad, USA). T3 homozygous seedlings of two transgenic lines were used for analysis.

### Phenotype Analysis of Transgenic *Arabidopsis* Plants

Phenotype analysis was performed as we described previously (Zhang et al., 2016b). Germination assays were carried out on three replicates of 50 seeds. Seeds were sterilized with 75% (v/v) ethanol solution for 1 min and with 2% (v/v) chlorine solution for 10 min, and then rinsed four to five times in sterile distilled water. The sterilized seeds were then sown on MS medium, and the plates were incubated at 4°C for 2 d in the dark before germination and were subsequently grown in a growth chamber at 23°C with 16/8 h light/dark photoperiod. Germination rates were scored at times with one day intervals within 10 d of incubation. Fifteen d-old seedlings grown on MS medium were used to determine root length, hypocotyl length and seedling height. Twenty-eight d-old plants were used to measure rosette leaf number. Flowering required time was recorded from 10 plants from each line when the inflorescence grew to 1 cm. Twenty-two d-old plants were used to determine leaf growth rates during a one week period when plants were incubated under normal (23°C) or chilling (0°C) temperature growth conditions.

### Chilling Tolerance Test of Transgenic *Arabidopsis* Plants

To determine chilling tolerance of the transgenic plants, a cold treatment assay was performed as described previously (Wang et al., 2018b). Twenty-two d-old *Arabidopsis* plants from WT and transgenic plants were used to test chilling tolerance. Plants were subjected to chilling stress for 6 days at 0°C with 16/8 h light/dark photoperiod. *Fv/Fm* and EL were measured at one day intervals during chilling condition. The cold treatment experiment was performed in triplicate.

### Determination of ROS Accumulation and antioxidant enzyme activities

ROS accumulations and antioxidant enzyme activities in *Arabidopsis* plants were measured following the method described previously (Wang et al., 2018b). The excised *Arabidopsis* leaves were incubated in a 1 mg/ml nitro blue tetrazolium (NBT) solution (pH 3.8) or in a 1 mg/ml diaminobenzidine (DAB) solution (Sigma, Germany) for 8 h in the dark to determine the localization of hydrogen peroxide (H_2_O_2_) and superoxide radicals (O_2_^•−^), respectively. H_2_O_2_ concentration was assayed by monitoring the absorbance of the titanium-peroxide complex at 415 nm and the nitrite formation from hydroxylamine in the presence of O_2_^•−^ at 530 nm. The determined H_2_O_2_ and O_2_^•−^ concentrations were expressed on fresh weight basis as μmol/g FW. Superoxide dismutase (SOD) activity was assayed by measuring the reduction of nitroblue tetrazolium chloride (NBT) at 560 nm. Catalase (CAT) activity was assayed by measuring the initial rate of H_2_O_2_ decomposition at 240 nm in a reaction with 10 mM H_2_O_2_. SOD and CAT activities were calculated and expressed on fresh weight basis as U/g FW. The experiments were performed in triplicate.

### Statistical Analysis

The experiments were completely randomized designs. All data are presented as the means ± standard error ( *± SE*) of at least three replicates. Statistical analyses of two groups were performed by student’s t-test and significant differences were indicated by “**”(P ≤0.01) or “*” (P ≤0.05), while statistical comparisons between more than three groups were performed by one-way analysis of variance (ANOVA) and significant differences (P ≤ 0.05) were indicated by different letters above bars.

## Results

### Expression of *CsDDI1*, *DDR1*, and *DDB1* Genes in Response to Cold Acclimation

**Figure 1:**
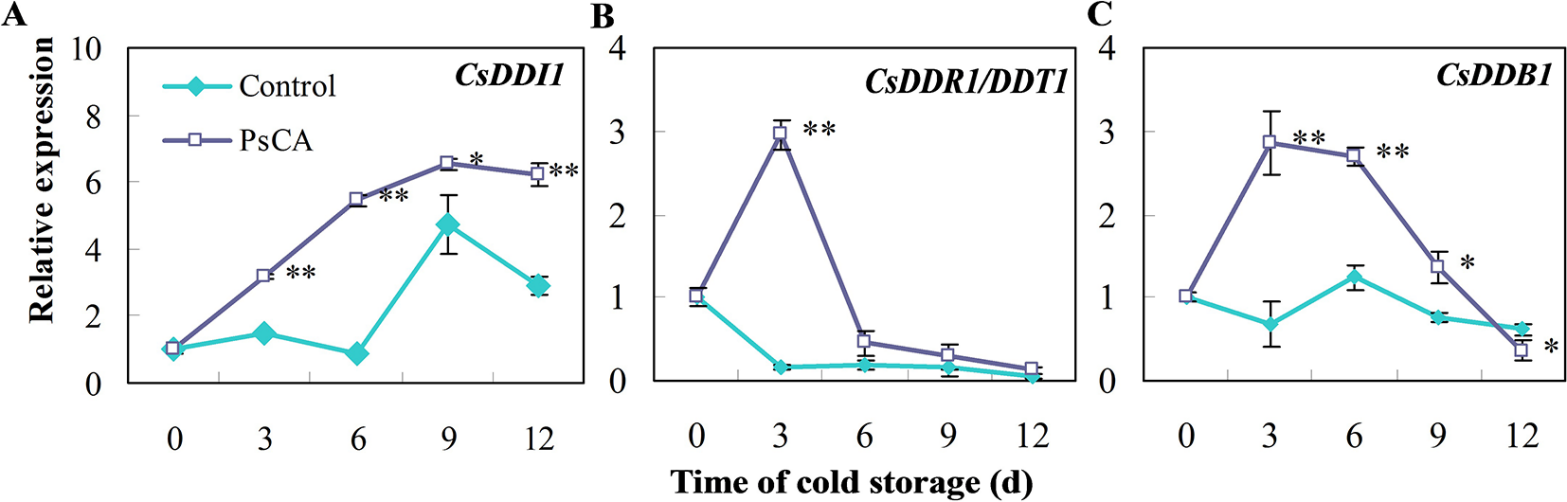
Effects of pre-storage cold acclimation (PsCA) on relative expression of three DNA damage- or repair-related genes in cold-stored cucumber. **(A)**, relative expression of *CsDDI1*; **(B)**, relative expression of *CsDDR1*/*DDT1*; **(C)**, relative expression of *CsDDB1*. Fruit were either directly placed at 5°C (Control) or were first incubated at 10°C for 3 d and then stored at 5°C (PsCA). The relative expression was evaluated by quantitative real-time PCR (qRT-PCR) using gene-specific primers (**Table S1**) and the expression data were all normalized to 100% (1.0) at 0 d of the control. Significant differences between the control and PsCA are indicated by “**”(P ≤ 0.01) or “*” (P ≤ 0.05). Data are presented as means ± standard errors ( ± *SE*) (n = 3).

We investigated expression patterns during pre-storage cold acclimation (PsCA) treatment and cold storage for DDI1, DDR1 and DDB1, which are all involved in DNA repair responses (Al Khateeb and Schroeder, 2009; Fujimori et al., 2014; Maric et al., 2017). In fruit exposed to cold stress from the very beginning (the control), *CsDDI1* expression remained almost unchanged until 6 d, while that of the PsCA-treated cucumber significantly increased following 3 d of cold acclimation and kept increasing even after the fruit were placed in chilling stress condition (**Figure 1A**). As for *CsDDR1* and *CsDDB1,* they did not show obvious increased expression in fruit exposed to the control treatment, but were highly up-regulated following 3 d of PsCA treatment. However, the expression decreased dramatically when the fruit were removed to cold stress (**Figures 1B, C**). These results suggest that PsCA triggered a kind of long-term expression for *CsDDI1* in cold stress, but not for *CsDDR1* and *CsDDB1.* Therefore, further experiments were conducted to address the role of *CsDDI1* in chilling tolerance.

### Biosynthesis of Both ABA and NO are Involved in Cold Acclimation

**Figure 2:**
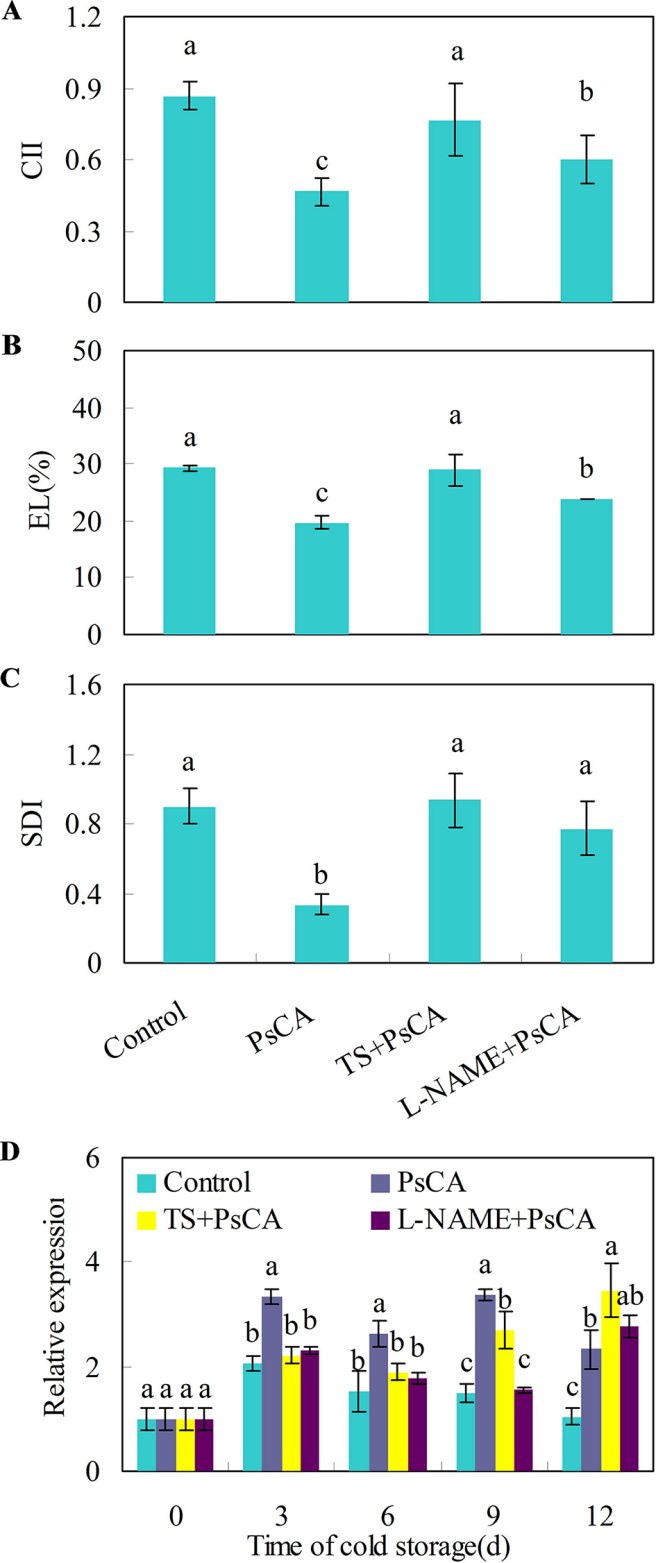
Effects of L-NAME and TS on chilling tolerance and *CsDDI1* expression as affected by PsCA in cold-stored cucumber. For the control treatment, fruit were directly placed at 5°C. For PsCA treatment, fruit were first incubated at 10°C for 3 d and then stored at 5°C. For the application of the combination treatments, the fruit were first sprayed with TS at 50 μM or L-NAME at 100 μM and then air-dried at ambient temperature for 3 h before exposure to cold acclimation at 10°C for 3d. Following cold acclimation, the fruit were then placed 5°C for 12 d. Chilling injury indices (CII) **(A)** and electrolyte leakage (EL) **(B)** were evaluated after storage at 5°C for 12 d. Secondary disease indices (SDI) **(C)** were evaluated after the cucumbers were transferred to 20°C following 12 d of storage at 5°C. The relative expression of *CsDDI1* **(D)** was evaluated by quantitative real-time PCR (qRT-PCR) using specific primers (**Table S1**) and the expression data were all normalized to 100% (1.0) at 0 d of the control. L-NAME, L-nitro-arginine methyl ester, nitric oxide biosynthesis inhibitor, TS, tungstate, ABA biosynthesis inhibitor. Significant differences between the control and treatments are indicated by different letters above each bar (P ≤ 0.05). Data are presented as means ± standard errors ( ± *SE*) (n = 3).

Cold acclimation induces endogenous ABA and NO accumulation, which are positively related to chilling tolerance in *Arabidopsis* plants (Cuevas et al., 2008; Zhao et al., 2009). To investigate whether endogenous NO and ABA accumulation is necessary for chilling tolerance induced by cold acclimation, TS (tungstate, an ABA biosynthesis inhibitor) and L-NAME (L-nitro-arginine methyl ester, a nitric oxide biosynthesis inhibitor) were applied to cucumber fruit before exposure to cold acclimation. Compared with the control treatment, pre-storage cold acclimation (PsCA) significantly reduced chilling injury index (CII), electrolyte leakage (EL) and secondary disease index (SDI) (**Figures 2A–C**), suggesting PsCA enhances strong chilling tolerance in cucumber fruit. However, the application of TS or L-NAME significantly aggravated chilling injury, as was reflected by increased CII, EL and SDI relative to PsCA treatment (**Figures 2A–C**). This strongly indicates that PsCA-induced chilling tolerance involves biosynthesis of endogenous ABA and NO.

Measuring the expression levels of *CsDDI1* after these four treatments confirmed that *CsDDI1* is expressed at significantly higher levels after PsCA than the control treatment, in agreement with the results in **Figure 1A**. However, compared with PsCA treatment, application of L-NAME or TS before cold acclimation reduced *CsDDI1* expression for up to 9 d of exposure to cold stress (**Figure 2D**), suggesting that ABA and NO are involved regulating expression of *CsDDI1*.

### H_2_O_2_ Plays Crucial Roles in Initiating Cold Acclimation

**Figure 3:**
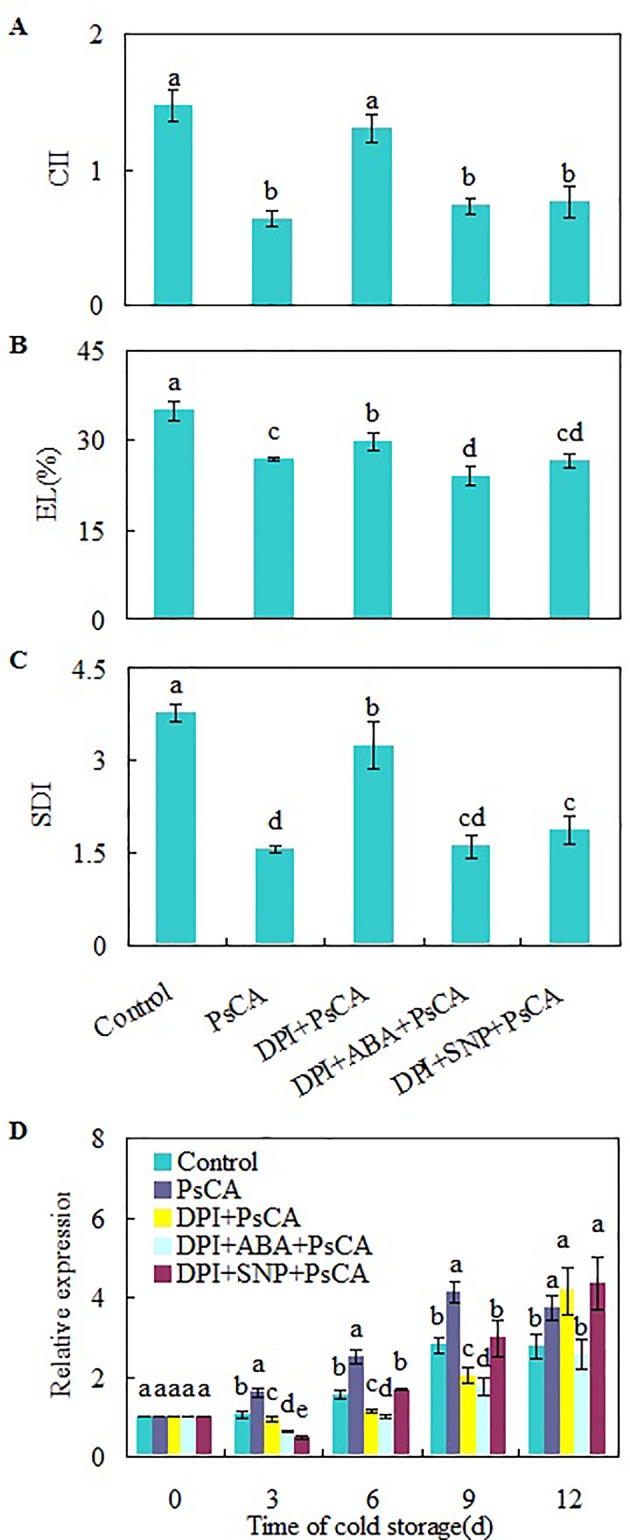
Regulation of endogenous H_2_O_2_, NO and ABA on chilling tolerance and *CsDDI1* expression of cold-stored cucumbers. For the control treatment, fruit were directly placed at 5°C. For PsCA treatment, fruit were first incubated at 10°C for 3 d and then stored at 5°C. For the application of the combination treatments, the fruit were first sprayed with DPI, incubated in plastic bags for 3 h, air-dried at ambient temperature before ABA (at 100 μM) or SNP (at 10 μM) was applied. After all reagents were sprayed, then the fruit were cold acclimated at 10°C for 3 d before being finally placed at 5°C for 12 d. Chilling injury indices (CII) **(A)** and electrolyte leakage (EL) **(B)** were evaluated after storage at 5°C for 12 d. Secondary disease indices (SDI) **(C)** were evaluated after the cucumbers were transferred to 20°C following 12 d of storage at 5°C. The relative expression of *CsDDI1* **(D)** was evaluated by quantitative real-time PCR (qRT-PCR) using specific primers (**Table S1**) and the expression data were all normalized to 100% (1.0) at 0 d of the control. DPI, diphenylene iodonium, NADPH oxidase inhibitor; SNP, sodium nitroprusside, nitric oxide (NO) donor. Significant differences between the control and treatments are indicated by letters above each bar (P ≤ 0.05). Data are presented as means ± standard errors ( ± *SE*) (n = 3).

H_2_O_2_ is considered a central signaling molecule in plant responses to biotic and abiotic stresses (Yoshioka et al., 2003; Xia et al., 2009). As H_2_O_2_ generated by NADPH oxidase is involved rapid systemic signaling associated with responses to abiotic stresses (Miller et al., 2009; Ben Rejeb et al., 2015), to check the role of endogenous H_2_O_2_ generated at the early stage of cold acclimation, DPI (diphenylene iodonium), an NADPH oxidase inhibitor (Cross and Jones, 1986), was applied before cold acclimation. CII, EL, and SDI of DPI+PsCA treatment were not obviously lower than those of the control, but significantly higher than those of PsCA treatment alone (**Figures 3A–C**), suggesting that endogenous H_2_O_2_ generation is required for initiation of cold acclimation. Measuring the expression levels of *CsDDI1* again confirmed that PsCA enhances expression of *CsDDI1* (**Figure 3D**). Furthermore, it showed that H_2_O_2_ biosynthesis inhibition before cold acclimation (DPI+PsCA) significantly reduced expression of *CsDDI1* on 3 d, 6 d and 9 d, suggesting that H_2_O_2_ is involved in cold acclimation.

To further confirm the involvement of H_2_O_2_ in PsCA-induced defense against chilling stresses, we analyzed the expression of six more defense genes related to chilling resistance: *ASR1*, *GSH-Px*, *Prd-2B*, *SOD(Cu-Zn)*, *L-APX6* and *POD* (Yoshimura et al., 2004; Xia et al., 2017; Wang et al., 2018a). The results showed DPI application before cold acclimation strongly downregulated all the six genes compared to PsCA treatment alone (**Figure S6**). These results suggest that H_2_O_2_ is necessary during the process of cold acclimation.

To investigate the relationship between H_2_O_2_, ABA and NO, exogenous ABA or NO was applied following inhibition of endogenous H_2_O_2_ by DPI. Either ABA or SNP were capable of restoring chilling tolerance compromised by DPI (**Figures 3A–C**). However, *CsDDI1* gene expression was not restored by either compound (**Figure 3D**). In other words, when endogenous H_2_O_2_ is inadequate, neither ABA nor NO is sufficient to upregulate *CsDDI1* gene. This implies that H_2_O_2_ plays a crucial role in regulating *CsDDI1* expression and that the role of ABA and NO in regulating *CsDDI1* expression and cold acclimation was independent of H_2_O_2_, or the function of CsDDI1 might be complemented by other family members of DDI proteins that could be regulated by ABA or NO.

It is noted that TS+PsCA and L-NAME+PsCA treatments in **Figure 2** and DPI+PsCA in **Figure 3** had lower chilling tolerance but higher *CsDDI1* gene expression than PsCA treatment on 12 d. This raises the question about whether upregulation of *CsDDI1* gene during cold acclimation and early during cold stress really contributes to chilling tolerance. To address this question, full-length cDNA of *CsDDI1* was isolated and used to generate *Arabidopsis* lines overexpressing *CsDDI1*.

### Bioinformatics Analysis and Localization of *CsDDI1*

**Figure 4:**
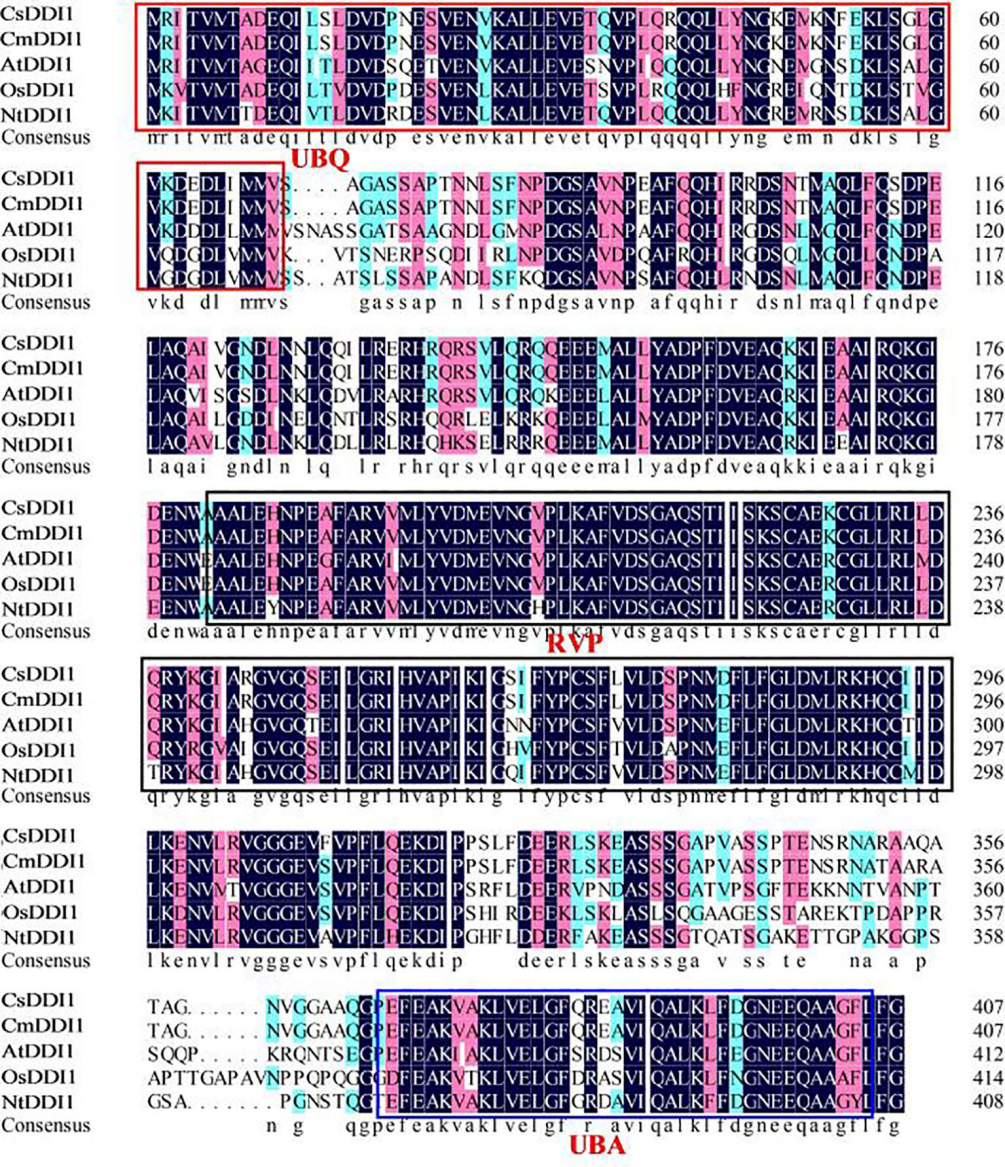
Multiple alignment of the amino acid sequences of CsDDI1s with DDI1s from four other plant species. Species names are abbreviated as follows: At, *Arabidopsis thaliana*; Os, *Oryza sativa*; Cs, *Cucumis sativus*; Cm, *cucumis melo*; Nt, *Nicotiana tabacum;* Sl, *Solanum lycopersicum*. Protein names and the corresponding Genbank accession numbers of the proteins are: CsDDI1 (XP_004139767.1), CmDDI1 (XP_008447792.1), AtDDI1 (AT3G13235), OsDDI1 (Os02g0198600), NtDDI1 (XP_016465827.1). The UBQ, RVP and UBA conserved domains are showed in the red, black and blue boxes, respectively.1.

Plant DNA damage inducible genes play a critical role in defense responses in a number of different plants. However, few *DDI1* genes have so far been identified in plants. To clone the *CsDDI1* gene from cucumber fruit, the coding sequence of *CsDDI1* was obtained from Cucumber Genomic database. The full length cDNA of *CsDDI1* contained an ORF of 1,224 bps coding for 15 exons separated by 14 introns (**Figure S1A**), as validated by PCR amplification and sequencing. The predicted ORF encodes a protein of 407 amino acid residues (**Figure S1B**) with an estimated MW of 44.76 kDa and a *pI* of 4.92 according to the computed *pI*/MW program. Multiple alignments of CsDDI1 and DDI1 proteins from five other plants including melon, *Arabidopsis*, rice, tomato and tobacco show shared sequence identities between 34.3% and 99.3% (**Table S2**) with Cucumber CsDDI1. CsDDI1 contains three conserved domains, UBQ (1–70 aa, ubiquitin homologues), RVP (181–304 aa, RP_DDI: retropepsin-like domain of DNA damage inducible protein) and UBA (369–405 aa, UBA/TS-N domain) (**Figure S1** and **Figure 4**), the typical domains of DDI1 proteins in living organisms (Nowicka et al., 2015).

**Figure 5:**
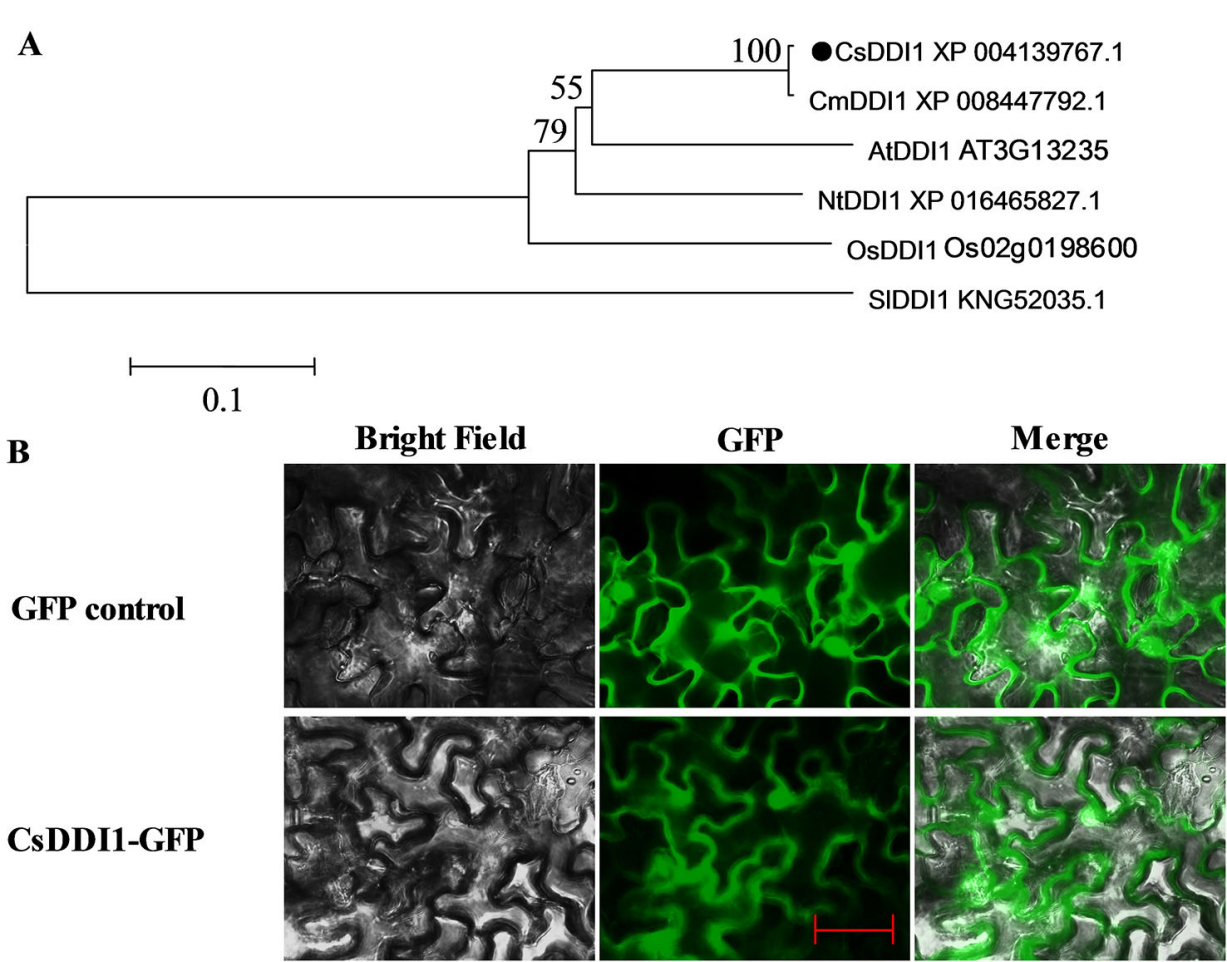
Phylogenetic analysis and subcellular localization of CsDDI1. **(A)** Phylogenetic tree based on comparison between protein sequences of CsDDI1 and DDI1 from five other plant species. The phylogenetic tree was produced using the Neighbor-Joining (NJ) method in the MEGA5 program. Species names are abbreviated as follows: At, *Arabidopsis thaliana*; Os, *Oryza sativa*; Cs, *Cucumis sativus*; Cm, *cucumis melo*; Nt, *Nicotiana tabacum;* Sl, *Solanum lycopersicum*. The accession numbers are indicated following protein name. CsDDI1 is marked by a black dot. **(B)** Subcellular localization of CsDDI1 in tobacco leaves. The coding sequence of *CsDDI1* without stop codon was cloned into a transient expression vector (*pCAMBIA2300-GFP*) driven by the CaMV 35S promoter. The fusion constructs and control vector were electroporated into *Agrobacterium tumefaciens* strain GV3101, which were then infiltrated into tobacco (*Nicotiana benthamiana*) leaves. After 72 h of the infiltration, GFP ﬂuorescence was imaged using a ﬂuorescence microscope. The length of the red bar is 50 μm.

A phylogenetic tree was reconstructed using the deduced amino acid sequence of CsDDI1 and five other plant DDI1 proteins, revealing they were clustered as one clade and CsDDI1 is most closely related to CmDDI1 from melon (**Figure 5A**). Transient expression of *CsDDI1* in tobacco leaf indicated that CsDDI1 proteins were distributed in the nucleus and cytoplasm of tobacco leaf cells (**Figure 5B**), suggesting that they may function to protect cytoplasmic and nuclear DNA.

### Phenotype of Transgenic *Arabidopsis* Plants Overexpressing *CsDDI1*

Two transgenic lines (OE1 and OE2) overexpressing the full-length of *CsDDI1* under the CaMV 35S promoter were generated (**Figures S2** and **S3A**). PCR analysis using DNA from T1 and T2 generations as templates confirmed that the *CsDDI1* gene was successfully transformed into *Arabidopsis* plants (**Figures S3B, C**). Semi-quantitative PCR, using cDNA from T3 generations as templates, confirmed that *CsDDI1* was stably expressed in *Arabidopsis* plant (**Figure S3D**).

The transgenic plants showed higher germination rate on 4 d and 6 d after been sown in the media (**Figure S4A**), higher root length (**Figure S4D**), more rosette leaves (**Figure S4E**) and shorter time required for flowering (**Figure S4F**) than the wild-types. Hypocotyl and seedlings of transgenic plants were longer than those of wild-types, although the differences were not statistically significant. (**Figures S4B, C**). Furthermore, transgenic plants showed faster leaf growth rates compared with wild-type plants (**Figures S4G, H**).

### *CsDDI1* Overexpression Confers Chilling Tolerance in *Arabidopsis* Plants

**Figure 6:**
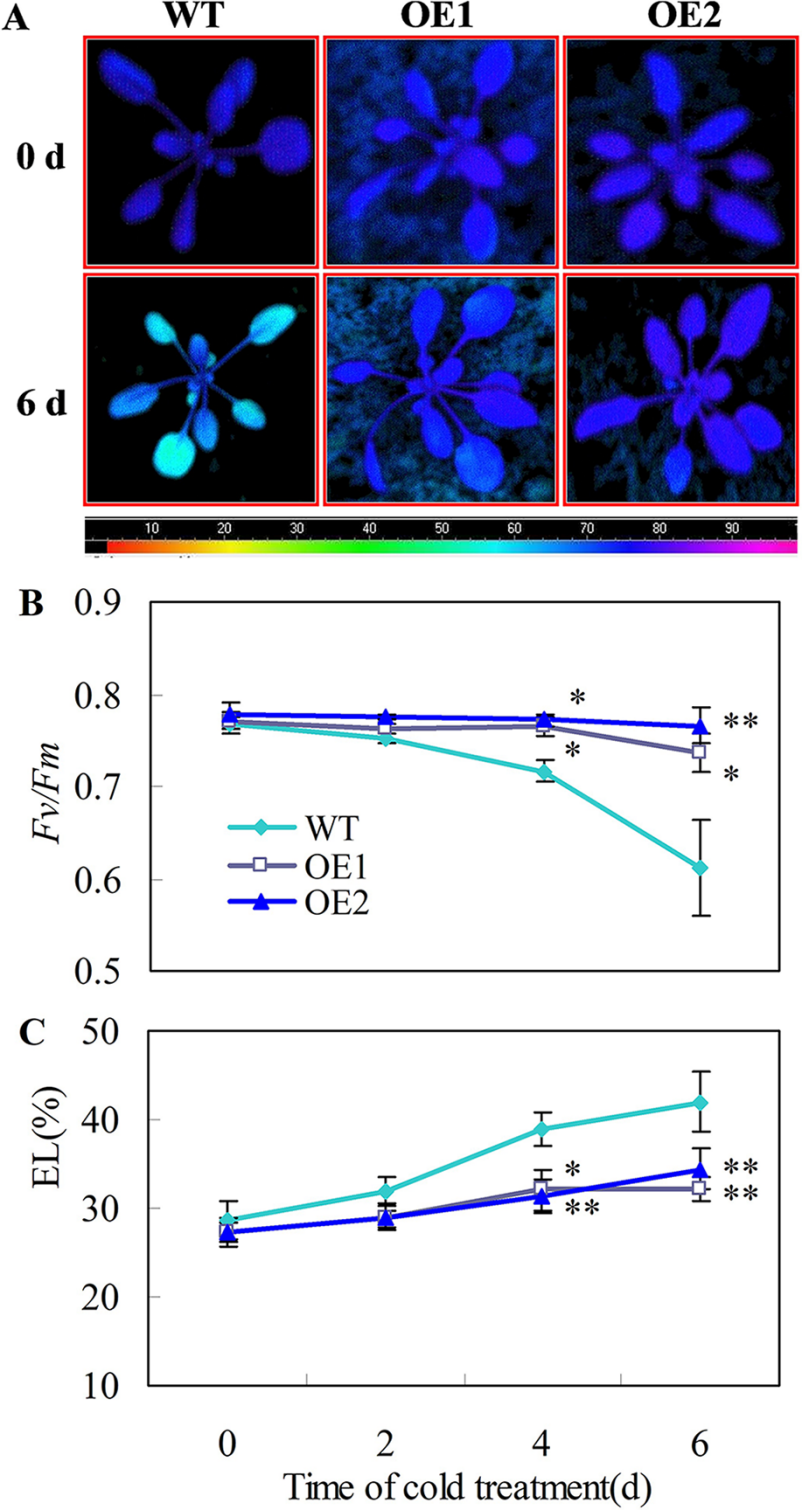
Transgenic *Arabidopsis* plants overexpressing *CsDDI1* display tolerance to chilling stress. **(A)** Images of chlorophyll fluorescence of chilling temperature (0°C)-treated leaves. Photographs show a representative picture of three repeated experiments. The color code depicted at the bottom of the image ranged from 0 (left) to 1.0 (right). **(B)** The maximum PSII quantum yield (*Fv/Fm*) values. **(C)** Electrolyte leakage (EL). WT, wild-type; OE1 and OE2, two transgenic lines over-expressing *CsDDI1*. For evaluating chilling tolerance of transgenic plants, 22-d old plants of the WT and *35S::CsDDI1* transgenic plants (OE1 and OE2) were subjected to 0°C for 6 d. Significant differences between the WT and OE lines are indicated by “**”(P ≤ 0.01) or “*” (P ≤ 0.05). Data are presented as means ± standard errors ( ± *SE*) (n = 3).

Chilling tolerance was assessed for 22 d-old *Arabidopsis* seedlings. When 22 d-old plants were subjected to 0°C for 6 days, the transgenic plants showed stronger chlorophyll fluorescence (**Figure 6A**), higher *Fv/Fm* ratios (**Figure 6B**) and lower EL (**Figure 6C**), than the wild-type plants. Moreover, no leaf expansion was observed in wild-type plants, whereas leaves on the transgenic lines continued to grow under chilling stress (0°C) (**Figures S4G, H**).

### *CsDDI1* Overexpression Enhanced Antioxidant Capacity in Transgenic *Arabidopsis* Lines under Chilling Stress

**Figure 7:**
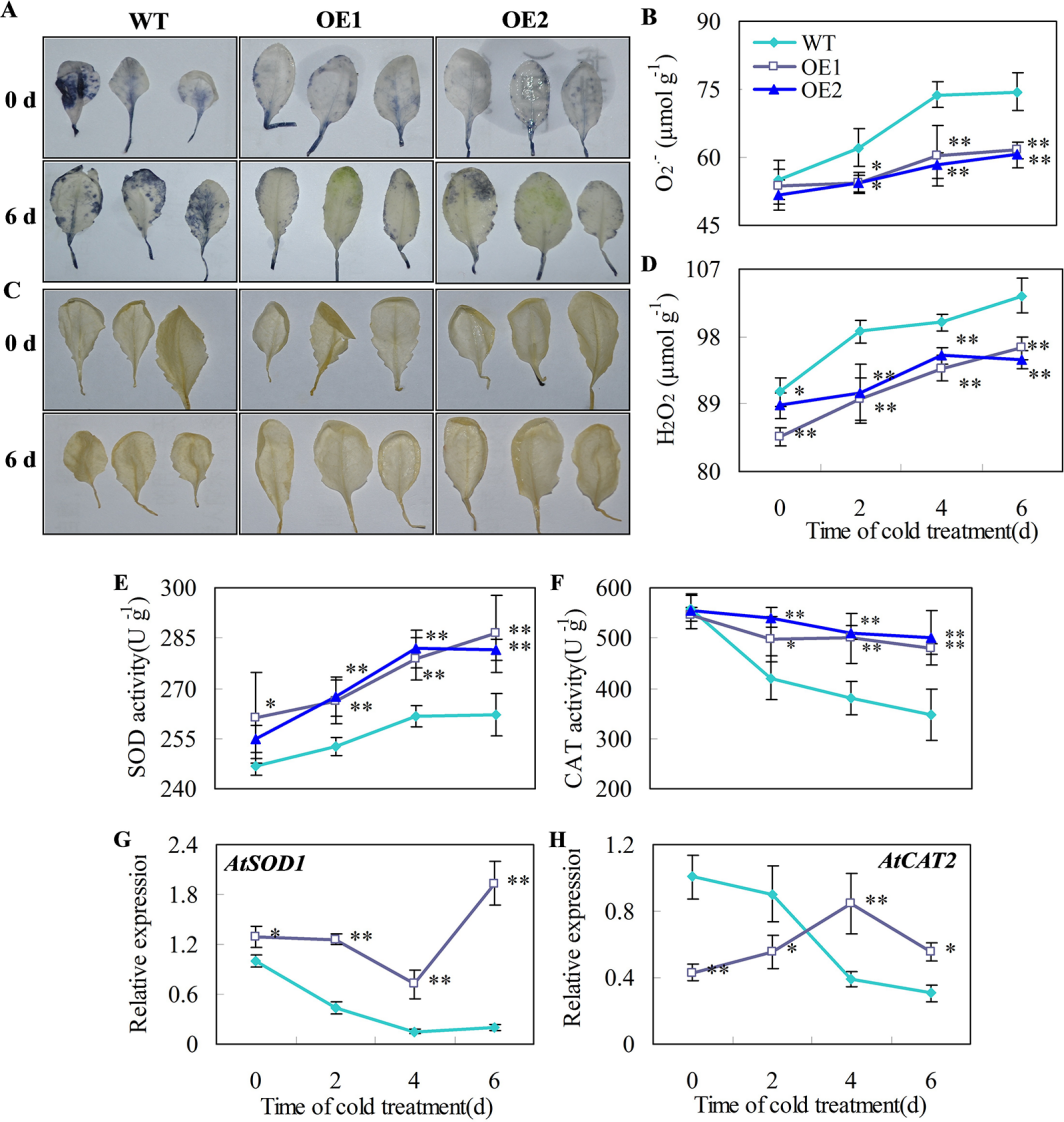
Effects of *CsDDI1* overexpression on O_2_^•−^ and H_2_O_2_ accumulations, and CAT and SOD expression and activities in *Arabidopsis* plants. Twenty-two d old seedlings were subjected to 0°C for 6 days. O_2_^.-^ location **(A)** and concentration changes **(B)** in *Arabidopsis* leaves were assayed with nitro blue tetrazolium (NBT). H_2_O_2_ location **(C)** and concentration changes **(D)** were assayed with diaminobenzidine (DAB). Superoxide dismutase (SOD) activity **(E)** was assayed by measuring the reduction of NBT. Catalase (CAT) activity **(F)** was assayed by measuring the initial rate of H_2_O_2_ decomposition. Gene expression of *AtSOD1* **(G)** and *AtCAT2* **(H)** were assayed by qRT-PCR. The gene expression data were normalized to 100% (1.0) at 0 d of the wild-type plants. Gene names and the corresponding AGI codes are: *AtSOD1*, AT1G08830 and *AtCAT2*, AT1G20630. WT, wild-type; OE1 and OE2, two transgenic lines overexpressing *CsDDI1*. Significant differences between the WT and OE lines are indicated by “**”(P ≤ 0.01) or “*” (P ≤ 0.05). Data are presented as means ± standard errors ( ± *SE*) (n = 3).

O_2_^•−^ and H_2_O_2_ are the major ROS in plants under chilling temperatures (Sharma et al., 2012; Del Río, 2015). Plant superoxide dismutase (SOD) and catalase (CAT) are part of the major ROS scavenging network (Ding et al., 2018). For example, decreases in the activities of SOD and CAT correlates with greater accumulation of O_2_^•−^ and H_2_O_2_ and higher chilling damage in cold-stored cucumber fruit (Wang and Zhu, 2017). Here, we showed that the accumulation of O_2_^•−^ and H_2_O_2_ in leaves of transgenic *Arabidopsis* lines overexpressing *CsDDI1* was reduced compared with the wild-type under chilling temperature (**Figures 7A–D**) and that the gene expression and enzyme activities of CAT and SOD were significantly enhanced in the transgenic plants (**Figures 7E–H**). These indicate that the transgenic plants overexpressing *CsDDI1* had higher antioxidant capacity than the wild-type plantlets.

### *CsDDI1* Overexpression Enhances Expression of Multiple Defense-Related Genes in *Arabidopsis* Under Chilling Stress

To explore how *CsDDI1* coordinates the regulation of chilling tolerance, the expression of nine genes involved in various defense pathways in response to cold stress were analyzed using qRT-PCR. The overexpression line 1 (OE1) was used for gene expression assay. The expression levels of *AtCOR47*, *AtCOR15b*, *AtPR1*, *AtHSP20*, *AtCML30*, *AtRD29A*, *AtNIA2*, *ATRH9*, and *AtPHR1* did not increase in wild-type plants, but all genes were highly upregulated in transgenic plants exposed to chilling stress (**Figure S5**).

## Discussion

Fruits, as reproductive organs, serve to provide the stable internal environment needed for seeds to develop and keep their genetic composition intact, even in adverse external environments. Therefore, the fruits are required to quickly respond to any environmental changes in order to stay physiologically healthy and genetically stable. Harvested fruits continue as living organs and play a critical role in protecting seeds inside (Wang et al., 2018a). Therefore, the fruits must have the capacity to initiate defenses against environmental stresses well before the onset of the real stress. Cold acclimation is a mechanism that can enable a plant or organ to gain tolerance to much more severe low temperature stress. We have previously showed that cold acclimation significantly reduces chilling injury in harvested cucumber fruit exposed to 5°C compared with the control (Wang et al., 2018a) and the results are confirmed in this study with two more independent sets of experiments (**Figures 2** and **3**), suggesting that cold acclimation enabled harvested fruit to adapt to chilling stress before the real chilling approaches.

Cold acclimation is a complex process that includes signal transduction and regulation of transcription (Thomashow, 1999). Studies have been focused on the role of the CBF (CRT/DRE-binding factor) pathway in the acquisition of cold tolerance (Park et al., 2015). However, it remained unclear whether DNA damage inducible proteins are involved in cold acclimation, as cold acclimation is normally initiated at critical temperatures which do not cause chilling injury. In this study, *CsDDI1* expression in the control fruit remained largely unchanged until the 6th day in cold stress (**Figure 1A**), but was highly increased in PsCA-treated cucumber following 3 d of cold acclimation (**Figure 1A**) and remained higher than the control during the remaining period in cold stress. Two more lines of evidences largely confirmed this trend (see **Figures 2** and **3**). These suggest that *CsDDI1* is involved in cold acclimation. In this study, the expression patterns of three genes involved in DNA repair pathways were induced by PsCA (**Figure 1**), implying non-chilling-stress temperature activated DNA repair responses. However, it is worth noting that, after cucumber fruit were transferred to chilling stress condition, *CsDDI1* expression continued to increase, while the transfer caused a sudden drop in *CsDDR1/CsDDT1* and *CsDDB1* transcription, which stayed at very low levels during cold stress (**Figures 1B, C**), implying *CsDDI1* could play a role in PsCA-induced chilling tolerance, but *CsDDR1/CsDDT1* and *CsDDB1* might not.

Recognition of cold signals lead to increased biosynthesis and accumulation of H_2_O_2_, NO and ABA (Cuevas et al., 2008; Zhao et al., 2009; Zhou et al., 2012; Xia et al., 2015), the early generation of which are required to trigger defense responses in plants (Rejeb et al., 2015; Chan et al., 2016; Yao et al., 2017). We conducted two sets of experiments to explore how these signal molecules are involved in regulation of cold acclimation in relation to *CsDDI1* expression. In the first one, inhibition of biosynthesis of either ABA or NO before PsCA treatment significantly aggravated chilling injury of cucumber compared with PsCA treatment alone, suggesting ABA and NO are necessary for initiating cold acclimation of cucumber fruit. In addition, inhibition of endogenous ABA or NO biosynthesis reduced *CsDDI1* transcription except for the last day (**Figure 2**), suggesting ABA or NO is necessary for activating transcription of *CsDDI1.*

In the second experiment, inhibition of H_2_O_2_ generation before PsCA treatment significantly reduced chilling tolerance and *CsDDI1* gene expression except on the last day relative to PsCA alone (DPI+PsCA vs PsCA, **Figure 3**), suggesting H_2_O_2_ is required for initiating cold acclimation and for activating *CsDDI1* expression. However, addition of ABA or NO after inhibition of H_2_O_2_ restores chilling tolerance, but did not restore *CsDDI1* expression levels to that of PsCA alone (DPI+ABA+PsCA and DPI+SNP+PsCA, **Figure 3**). Considering that inhibition of NO or ABA downregulated *CsDDI1* (see TS+PsCA and L-NAME treatments in **Figure 2D**), the results suggest that H_2_O_2_ is required for NO and ABA to induce *CsDDI1* expression and that chilling tolerance restored by ABA or NO was not dependent on DNA damage response. These results together suggest that H_2_O_2_ plays a crucial role in activating transcription of *CsDDI1*. It could be that the H_2_O_2_ induced by cold acclimation may serve as DNA damage signal, as ROS may cause DNA damage in plants (Roldan-Arjona and Ariza, 2009). Therefore, that DPI+ABA+PsCA or DPI+SNP+PsCA treatment did not upregulate *CsDDI1* could be resulted from the fact that DPI quenched the DNA damage signal.

To further study the role of *CsDDI1* in cold acclimation-induced chilling tolerance, we cloned the full-length cDNA and generated *Arabidopsis* plant lines overexpressing *CsDDI1*. The deduced CsDDI1 protein contains ubiquitin-like domains at its C and N termini and a retropepsin-like domain (RVP) (**Figure 4** and **Figure S1**), which are typical domains of DDI proteins involved in an ubiquitin-dependent pathway (Nowicka et al., 2015). It has been shown that DDI1 interacts with Ub through the UBA domain (Nowicka et al., 2015). Here we show that CsDDI1 protein was distributed in the nucleus and cytoplasm (**Figure 5**), implying CsDDI1 could play a role in DNA repair, as in yeast and mammals, ubiquitination have been discovered to be involved in DNA repair (Tian and Xie, 2013).

In eukaryotic organisms, ubiquitin is a small 8.5 kDa regulatory protein. Ubiquitination, the addition of ubiquitin to a substrate protein, mainly affects protein stabilization, including protein degradation, cellular location, activity, and interactions (Glickman and Ciechanover, 2002; Mukhopadhyay and Riezman, 2007). Moreover, ubiquitination plays important roles in the regulation of the cell cycle, stress tolerance, phytohormone levels, and cell differentiation (Lyzenga and Stone, 2012; Dametto et al., 2015). Overexpression or knockdown of *DDI1* in tomato plants did not show an aberrant developmental phenotype (Miao et al., 2014), while transgenic tobacco lines overexpressing a wheat ubiquitin gene (Ta-Ub2) showed earlier germination and enhanced growing (Guo et al., 2008).

In this study, two lines of transgenic *Arabidopsis* plants showed very similar phenotypes. Overexpression of *CsDDI1* in *Arabidopsis* showed earlier germination, faster growth and earlier flowering compared with the wild-type plants (**Figures S4A–F**). This implies that *CsDDI1* has similar function as ubiquitin, which enables the fruits and seeds to get mature faster in chilling stress by enhancing cell cycle.

Overexpression of *Ta-Ub2* in tobacco resulted in high tolerance to drought (Guo et al., 2008) and the deletion of *AtDDI1* increased susceptibility to pathogenic bacteria (Ding et al., 2016). In this study, transgenic *Arabidopsis* lines overexpressing *CsDDI1* showed higher *Fv/Fm* ratios (**Figures 6A, B**), lower EL (**Figure 6C**), and higher leaf growth rate (**Figures S4G, H**). These results, together with the results that PsCA upregulated *CsDDI1*, and TS, L-NAME and DPI downregulated *CsDDI1*, strongly suggest that *CsDDI1* positively regulate chilling tolerance of harvested cucumber fruit.

Antioxidant defense machinery is an important mechanism against abiotic stresses in plants (Asada, 2006; Miller et al., 2010). Accumulation of ROS under abiotic stress is regarded as inducer of DNA damage, such as double strand breaks, base deletion, and base modification (Jahnke et al., 2010; Sharma et al., 2012; Yao et al., 2013). These lead to increased homologous recombination and mutation frequency in plants under stress conditions (Shim et al., 2018). In plants, the major forms of ROS include superoxide radical ion (O_2_^•−^) and hydrogen peroxide (H_2_O_2_) (Mittler, 2002; Sharma et al., 2012; Farnese et al., 2016). The antioxidant enzymes, such as SOD and CAT, are crucial for ROS scavenging and maintenance of cell integrity (Asada, 2006; Xu et al., 2010). The increase in *SOD* and *CAT* gene expression and enzyme activities are usually related to enhanced stress tolerance in plants (Ding et al., 2018). In this study, *Arabidopsis* plants overexpressing *CsDDI1* displayed lower ROS levels (**Figures 7A–D**), and higher *CAT* and *SOD* gene expression and enzyme activities than the wild type plants under chilling stress condition (**Figures 7E–H**). These results indicate that *CsDDI1* overexpression lines have stronger antioxidant capacity and thereby decreased chilling-induced oxidative damage, which may help CsDDI1 play the role in repairing DNA.

It is noted that *AtCAT2* gene expression was not corrected to CAT activities (**Figures 7F, H**), as when CAT activities for both wild type and transgenic plants declines under chilling-stress condition, only did *AtCAT2* gene expression for wild type plants decline, while that for transgenic plants overexpressing *CsDDI1* demonstrated an increasing trend during the first 4 d under the same condition. Two reasons could be proposed for this. Firstly, ectopic expression of *CsDDI1* changed *AtCAT2* expression pattern, which contributed to the higher enzyme activity of CAT (**Figure 7F**). Secondly, different members of catalase gene family complement each other to form sufficient antioxidant capacity at different stages of plant growth or under different abiotic conditions. Therefore, although levels of *AtCAT2* expression was lower in the transgenic than in wild type plants during the first 2 d (**Figure 7H**), other member of CAT family could complement this and contribute to the higher enzymes activity for this period (**Figure 7F**). This implies that apart from *AtCAT2*, other *AtCAT* genes could also be activated by overexpression of *CsDDI1* in *Arabidopsis*. Work remains to be done to further investigate this.

Now the question is: how can CsDDI1 regulate the antioxidant defense system? The answer may lie in two independent lines of evidence. First, *Arabidopsis* overexpressing *CsDDI1* had significantly higher *Arabidopsis PR1* (*AtPR1*) gene expression than wild type following 6d of cold treatment (**Figure S5**). It is well documented that the onset of systemic acquired resistance (SAR) is associated with increased endogenous levels of salicylic acid (SA) (Malamy et al., 1990), and exogenous SA application also induces SAR and PR gene expression (Ward et al., 1991). Therefore, this study implies that *CsDDI1* overexpression *Arabidopsis* plants could be high in SA levels. SA significantly increases the activities of antioxidant enzymes in wheat seedlings (Agarwal et al., 2005). Exogenous SA reduces the excess H_2_O_2_ and enhances chilling tolerance of cucumber (*C. sativus* L.) seedings (Dong et al., 2014). Therefore, this work implies that DDI1 could activate antioxidant defense system by promoting SA biosynthesis through an as yet unknown mechanism.

In addition, expression of eight other *Arabidopsis* genes (*AtCOR47*, *AtCOR15b*, *AtHSP20*, *AtCML30*, *AtRD29A*, *AtNIA2*, *AtRH9*, and *AtPHR1*) were upregulated in transgenic *Arabidopsis* lines overexpressing *CsDDI1* under chilling stress condition (see **Figure S5**). These genes play roles in various defense pathways including cold acclimation, response to cold stress, ROS, RNA metabolism, and DNA damage repair (Wang et al., 2018b). These results suggest that *CsDDI1* positively regulates multiple defense responses which collaboratively contribute to the enhancement of chilling tolerance.

In conclusion, cold acclimation at 10°C significantly alleviated chilling injury of cucumber fruit stored in chilling stress conditions. There was little change in the expression of *CsDDI1* in the control fruit until severe chilling injury occurred, but expression was significantly upregulated right after cold acclimation. Application of NO, ABA or H_2_O_2_ inhibitors before exposure to cold acclimation downregulated *CsDDI1* expression and significantly aggravated chilling injury. When H_2_O_2_ generation was inhibited, the addition of NO or ABA restored chilling tolerance, but did not restore *CsDDI1* expression. These suggest that *CsDDI1* is involved in cold acclimation and that H_2_O_2_ plays a crucial role in activating transcription of *CsDDI1*. *Arabidopsis* lines overexpressing *CsDDI1* displayed faster growth in 23°C and stronger chilling tolerance in 0°C than wild-type plants. Additionally, they exhibited lower ROS levels and higher CAT and SOD expression and activity than the wild-type plants at 0 °C. Nine *Arabidopsis* genes involved in various defense responses were all upregulated in *Arabidopsis* plants overexpressing *CsDDI1*. These results strongly suggest *CsDDI1* plays a positive role in cold tolerance induced by cold acclimation in harvested cucumber fruit and that *CsDDI1* enhances the antioxidant system to scavenge ROS when upregulated.

## Data Availability Statement

All datasets for this study are included in the article/**Supplementary Material**.

## Author Contributions

SZ conceived and oversaw the work. BW performed the experiments and made the tables and figures. SZ, BW and GW wrote the manuscript. All authors have read and approved the manuscript.

## Conflict of Interest

The authors declare that the research was conducted in the absence of any commercial or financial relationships that could be construed as a potential conflict of interest.
