# Supplementary figures and images for: DNA Damage Inducible Protein 1 is Involved in Cold Adaption of Harvested Cucumber Fruit
Source: Front Plant Sci. 2020 Jan 24;10:1723. doi: 10.3389/fpls.2019.01723 (PMC6992665; doi:10.3389/fpls.2019.01723)

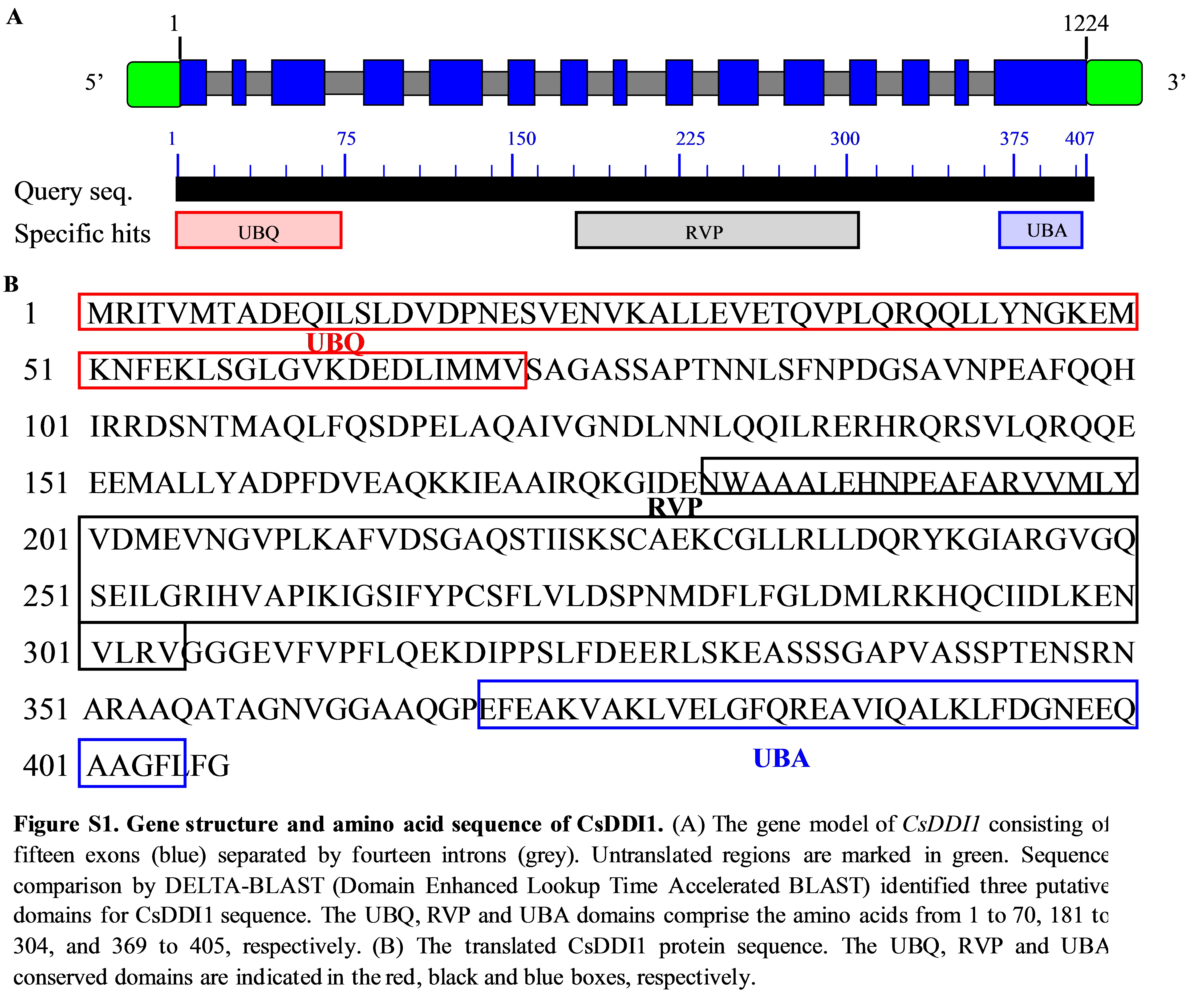

Supplement: Supplementary file 1 [file Image_1.jpeg]

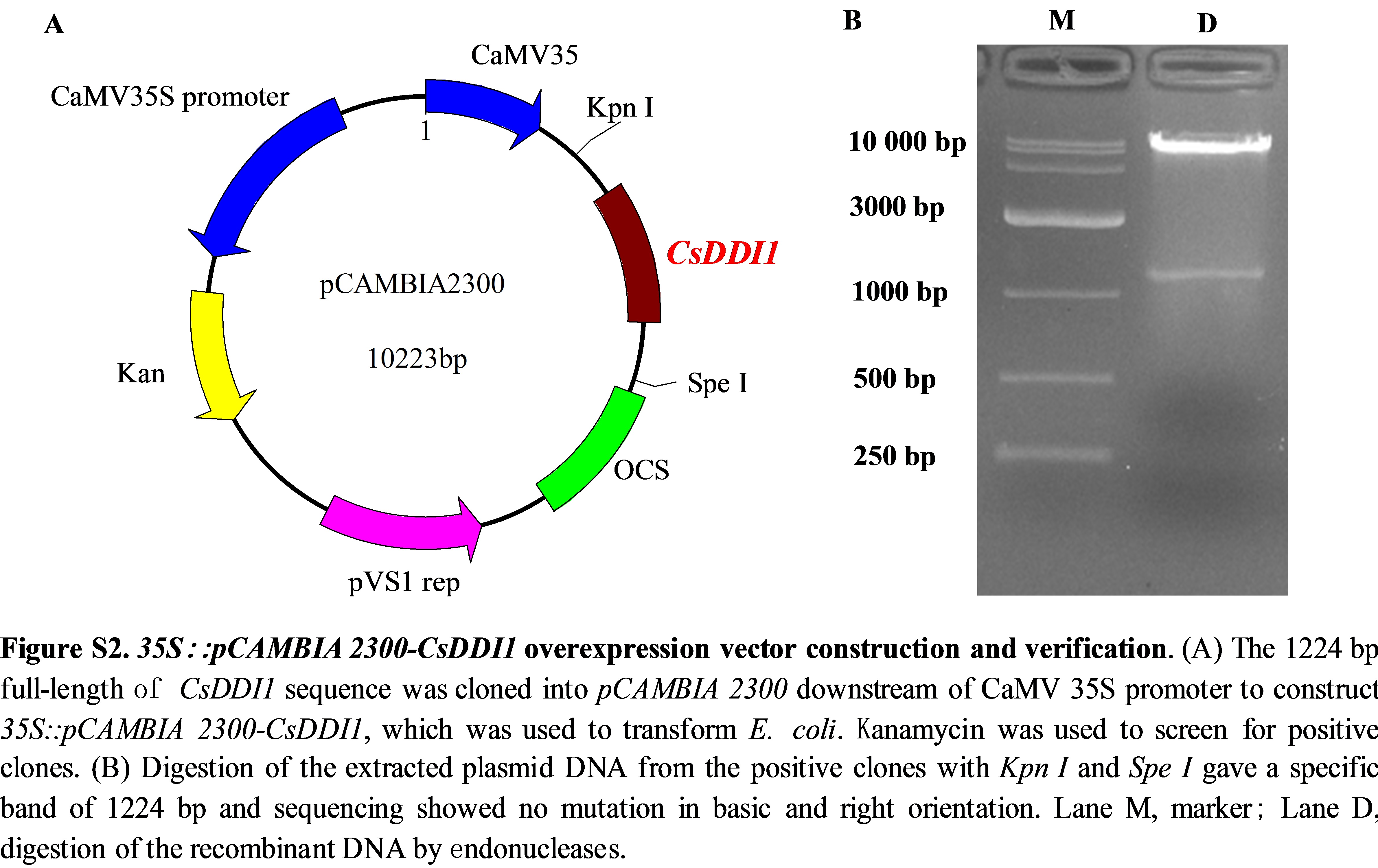

Supplement: Supplementary file 2 [file Image_2.jpeg]

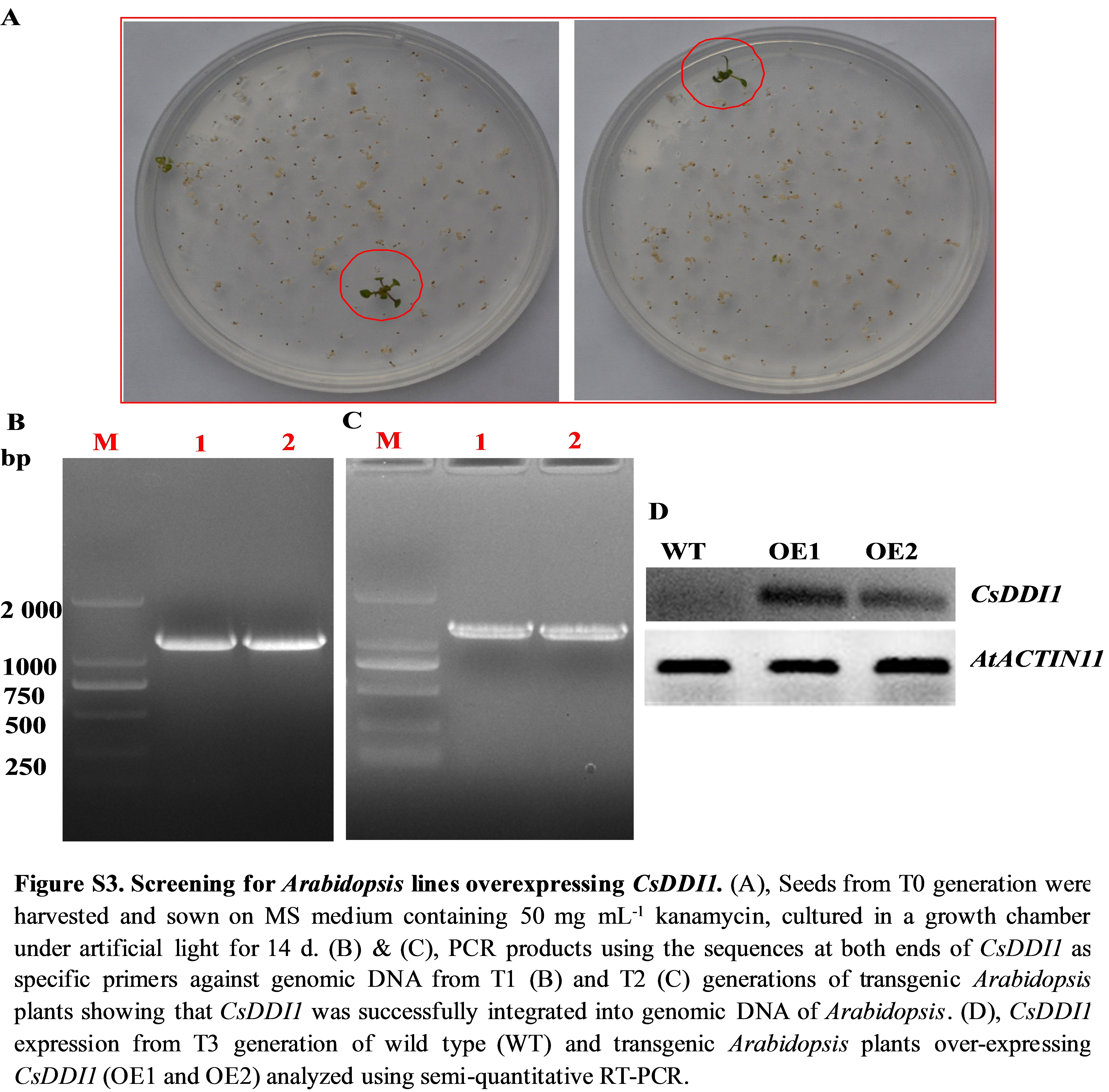

Supplement: Supplementary file 3 [file Image_3.jpeg]

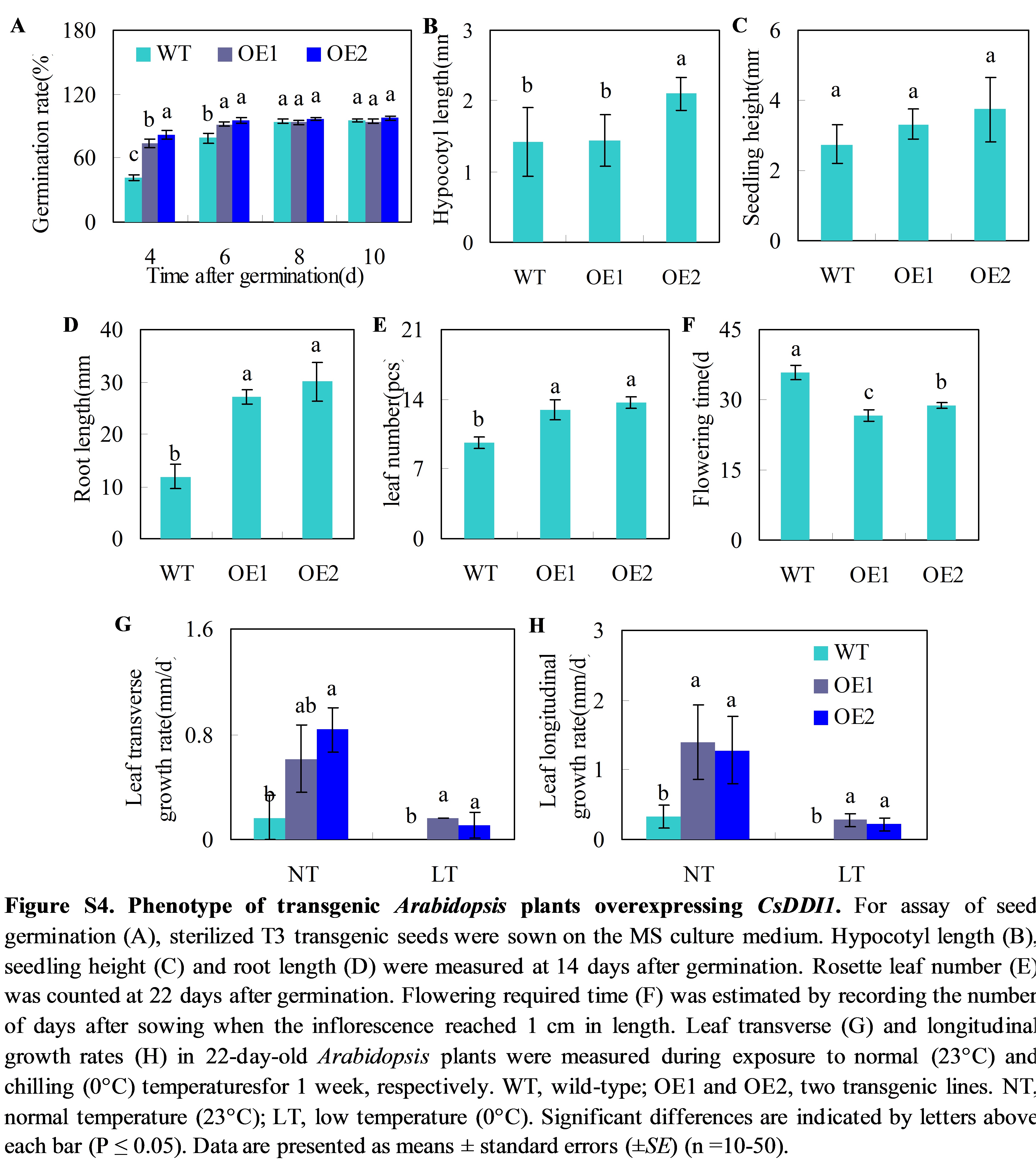

Supplement: Supplementary file 4 [file Image_4.jpeg]

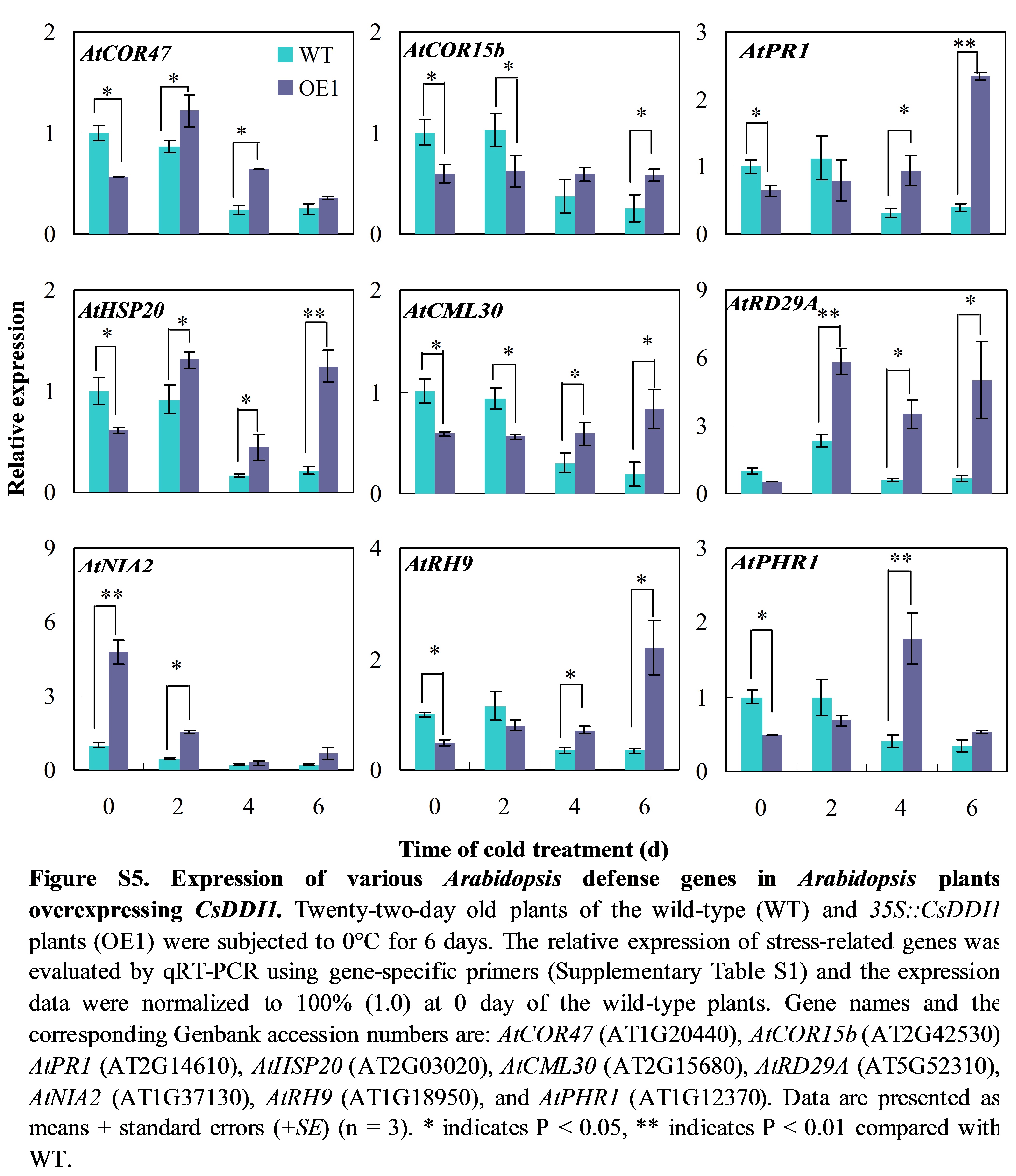

Supplement: Supplementary file 5 [file Image_5.jpeg]

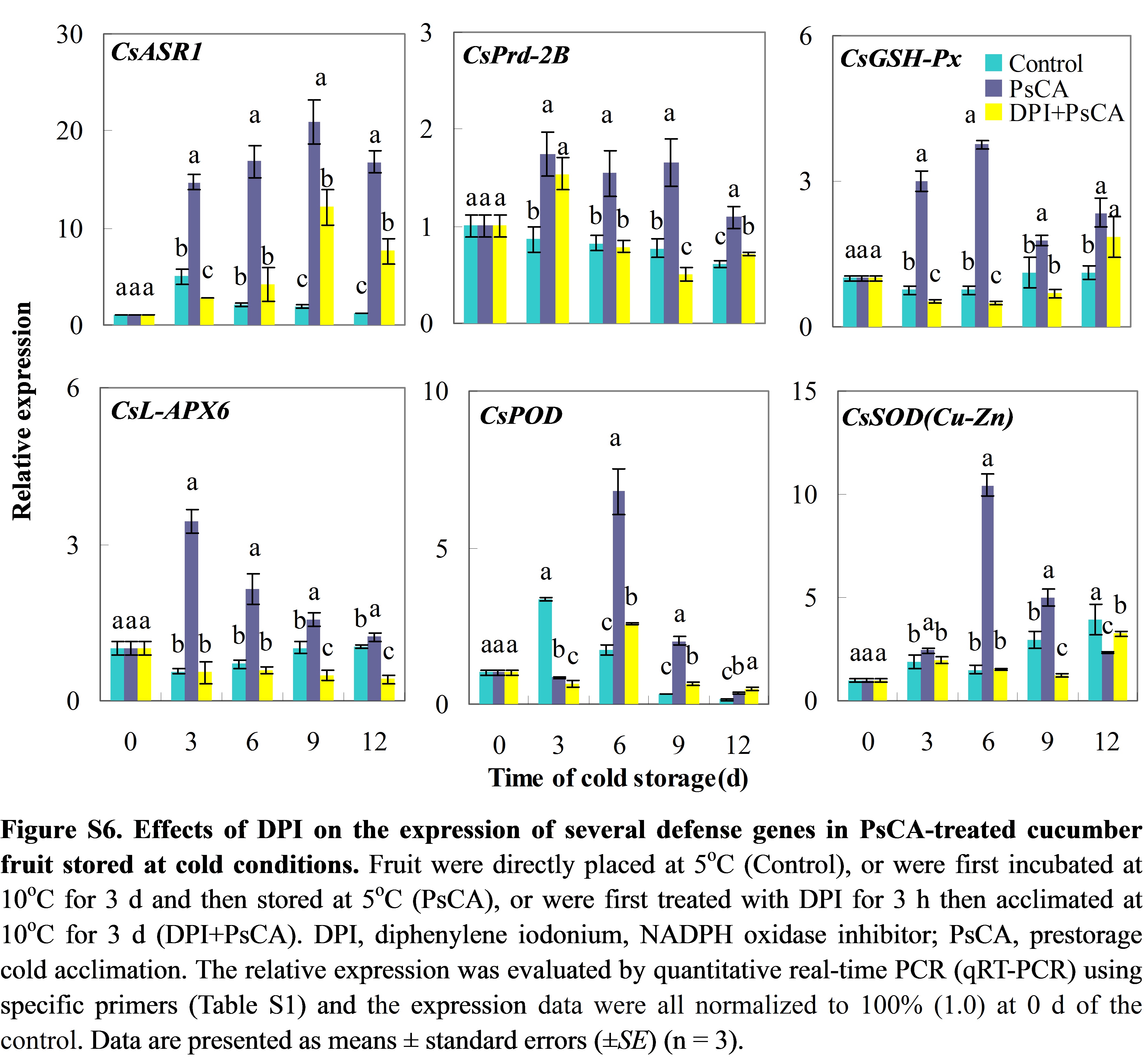

Supplement: Supplementary file 6 [file Image_6.jpeg]
