# Supplementary material for: DNA Damage Inducible Protein 1 is Involved in Cold Adaption of Harvested Cucumber Fruit
Source: Front Plant Sci. 2020 Jan 24;10:1723. doi: 10.3389/fpls.2019.01723 (PMC6992665; doi:10.3389/fpls.2019.01723)
Supplement: Supplementary file 7 [file Table_1.doc]

**Table S1. Specific primers used in PCR amplification.**

| Application | Primer sequences (5’–3’) | Amplicon | NCBI/AGI code | Restriction Site |
| --- | --- | --- | --- | --- |
| Full length cloning for *CsDDI1* | F1: GGGGTACCATGAGAATCACTGTGATGACAG | 1224 bp | XM_004139719.2 | *Kpn I* |
|  | F2: GGACTAGTTTCATCCAAAAAGAAACCCAGC |  |  | *Spe I* |
| Subcellular localization for *CsDDI1* | F3:GGGGTACCATGAGAATCACTGTGATGACAGC | 1221 bp | XM_004139719.2 | *Kpn I* |
|  | F4:GGACTAGTAAAAAGAAACCCAGCTGCCTG |  |  | *Spe I* |
| *CsActin* | F: AGGCCGTTCTGTCCCTCTAC | 150 bp | AB698859 |  |
|  | R: CAGTAAGGTCACGACCAGCA |  |  |  |
| *AtActin* | F: GCCAGTGGGCATTGGATTTG | 108 bp | AT3G12110 |  |
|  | R: CTCCAATCATGTGGTTCGGC |  |  |  |
| qPCR analysis for *CsDDI1* | F: TCACTGTGATGACAGCCGAC | 82 bp | XM_004139719.2 |  |
|  | R: AGCAGAGCCTTCACGTTCTC |  |  |  |
| qPCR analysis for *CsDDR1/DDT1* | F: CAGCGGCACAAAGGATGATG | 116 bp | XM_004134561.2 |  |
|  | R: TCCAGCACGCAAATCTGTCT |  |  |  |
| qPCR analysis for *CsDDB1* | F: ATGTGCAAACCCGTGGAGAT | 113 bp | XM_004135491.2 |  |
|  | R: ATTGTAGTCCCGGGCTCTCT |  |  |  |
| qPCR analysis for *AtCAT2* | F: CAAGTGTGGGGTGTCGAGAG | 117 bp | AT1G20630 |  |
|  | R: ACCCCAAAATGTCAAAACCAAGTT |  |  |  |
| qPCR analysis for *AtSOD1* | F: TCCTGAGATCACAAAGGCCAA | 101 bp | AT1G08830 |  |
|  | R: TCGCCTTCCTGGGTGAAAAA |  |  |  |
| qPCR analysis for *AtCOR47* | F: GACGTGTCTAATGGCCCACA | 96 bp | AT1G20440 |  |
|  | R: TAAAAGAGGTGGGAACGGGC |  |  |  |
| qPCR analysis for *AtCOR15b* | F: CAACTTGATGGCCGACCTCT | 84 bp | AT2G42530 |  |
|  | R: AGAAGAGTTTTCGTTGGTTCGT |  |  |  |
| qPCR analysis for *AtPR1* | F: CTCGGAGCTACGCAGAACAA | 87 bp | AT2G14610 |  |
|  | R: CGCTACCCCAGGCTAAGTTT |  |  |  |
| qPCR analysis for *AtHSP20* | F: CGTGGATGGAGGACGAGTTT | 119 bp | AT2G03020 |  |
|  | R: ATCGCCGGTGATTTCCACAA |  |  |  |
| qPCR analysis for *AtCML30* | F: TTTGAACGGCGATGGGAAGA | 96 bp | AT2G15680 |  |
|  | R: ACCATCCTGTTGCAGTCCTC |  |  |  |
| qPCR analysis for *AtRD29A* | F: ACGTTTGCTCCAAGTGGTGA | 79 bp | AT5G52310 |  |
|  | R: CCTCCAACGTTATCGGGGTC |  |  |  |
| qPCR analysis for *AtNIA2* | F: CATTTTCCTTTGCGCCACCA | 90 bp | AT1G37130 |  |
|  | R: AGCTCGAAGTAGCCAACCAC |  |  |  |
| qPCR analysis for *AtRH9* | F: GCCGTTACTCTGGTGGTTCA | 126 bp | AT1G18950 |  |
|  | R: CAAACCCACCAAAGCTGCTC |  |  |  |
| qPCR analysis for *AtPHR1* | F: TTGCCTTCCCTCCATTTTTCG | 101 bp | AT1G12370 |  |
|  | R: CTAATGTGCGCCGGTTATGC |  |  |  |
