# Supplementary material for: DNA Damage Inducible Protein 1 is Involved in Cold Adaption of Harvested Cucumber Fruit
Source: Front Plant Sci. 2020 Jan 24;10:1723. doi: 10.3389/fpls.2019.01723 (PMC6992665; doi:10.3389/fpls.2019.01723)
Supplement: Supplementary file 8 [file Table_2.docx]

**Table S2. Comparison of identity of cucumber DDI1 (CsDDI1) with DDI1 from five other plant species. Values indicate percentage identity, which were obtained by EMBOSS Stretcher (https://www.ebi.ac.uk/Tools/psa/emboss_stretcher/). Accession numbers are indicated in Figure 6A.**

|  | 1 | 2 | 3 | 4 | 5 | 6 |
| --- | --- | --- | --- | --- | --- | --- |
| 1: CsDDI1 | 100 |  |  |  |  |  |
| 2: CmDDI1 | 99.3 | 100 |  |  |  |  |
| 3: AtDDI1 | 76.5 | 77.1 | 100 |  |  |  |
| 4: OsDDI1 | 71.8 | 72.0 | 69.6 | 100 |  |  |
| 5: NtDDI1 | 77.3 | 77.3 | 75.3 | 71.2 | 100 |  |
| 6: SlDDI1 | 34.3 | 34.1 | 34.3 | 34.9 | 34.6 | 100 |
